# Whole genome sequencing of live attenuated *Leishmania donovani* parasites reveals novel biomarkers of attenuation and enables product characterization

**DOI:** 10.1038/s41598-017-05088-4

**Published:** 2017-07-05

**Authors:** Sreenivas Gannavaram, John Torcivia, Lusine Gasparyan, Amit Kaul, Nevien Ismail, Vahan Simonyan, Hira L. Nakhasi

**Affiliations:** 1 Division of Emerging and Transfusion Transmitted Diseases, Center for Biologics Evaluation and Research, US Food and Drug Administration, Silver Spring, MD 20993 USA; 2 High Performance Integrated Virtual Environment, Center for Biologics Evaluation and Research, US Food and Drug Administration, Silver Spring, MD 20993 USA; 3 DNA-HIVE, Rockville, 20850 MD United States

## Abstract

No licensed human vaccines are currently available against leishmaniasis. Several anti-leishmanial vaccines are currently undergoing testing, including genetically modified live-attenuated parasite vaccines. Studies with live attenuated *Leishmania* vaccines such as centrin deleted *Leishmania donovani* parasites (*LdCen* ^−/−^) showed protective immunity in animal models. Such studies typically examined the biomarkers of protective immunity however the biomarkers of attenuation in the parasite preparations have not received adequate attention. As several candidate vaccines enter clinical trials, a more complete product characterization to enable maintenance of product quality will help meet regulatory requirements. Towards this goal, we have determined the complete genome sequence of *LdCen* ^−/−^ and its parent strain Ld1S-2D (*LdWT*) and characterized the *LdCen* ^−/−^ vaccine strain using bioinformatics tools. Results showed that the *LdCen* ^−/−^ parasites, in addition to loss of the centrin gene, have additional deletions ranging from 350 bp to 6900 bp in non-contiguous loci on several chromosomes, most commonly in untranslated regions. We have experimentally verified a subset of these adventitious deletions that had no impact on the attenuation of the *LdCen* ^−/−^ parasites. Our results identified hitherto unknown features of attenuation of virulence that could be used as markers of product quality in production lots and highlight the importance of product characterization in parasitic vaccines.

## Introduction

Visceral leishmaniasis (VL) is a serious global public health problem caused by the infection of blood borne protozoan parasites of *Leishmania donovani/infantum/chagasi* complex. VL is one of the most deadly parasitic diseases and disproportionately affects the poorest and most vulnerable populations. An estimated 200,000–400,000 people contract VL every year in developing countries^1^ (http://www.who.int/mediacentre/factsheets/fs375/en/). High prevalence of asymptomatic VL complicates the goals of VL elimination^2^. Vaccination can not only help in preventing VL, but also in reducing the burden imposed by asymptomatic VL cases. Despite many attempts, there are no effective prophylactic vaccines against VL. Several different kinds of vaccines, most commonly recombinant antigens in combination with adjuvants, have been experimentally tested and continue to be tested^3^. More recently, the relative ease of manipulation of the *Leishmania* genome enabled creation of genetically modified parasites by eliminating genes essential for virulence. Following these advances, several genetically modified live attenuated *Leishmania donovani/infantum* parasites have been developed as potential vaccines in recent years^4^. Of the several *L*. *donovani* gene deletion mutant parasites, centrin-deleted parasites (*LdCen* ^−/−^) have been shown to be highly immunogenic in experimental animal studies in mice, hamsters, dogs, and *ex vivo* human studies^5–8^.

For several anti-*Leishmania* vaccines, much work has been done to identify biomarkers of efficacy, mainly immunological characteristics induced due to vaccination in the immunized host. Typically this involved measuring a post challenge response *in vitro* by antigen restimulation studies^9, 10^. Similarly, studies with gene deleted live attenuated *L*. *donovani* parasites have shown immunological biomarkers of vaccine efficacy such as high IFN-γ/IL-10 and induction of multifunctional cytokine secreting CD4^+^ and CD8^+^ T cells^5, 7, 11^. While none have entered clinical trials yet, a few of the live attenuated parasites have shown highly promising levels of protection in the experimental infections indicating that some of these mutant parasites might enter clinical trials. As some of these live attenuated *Leishmania* parasites advance to clinical trials following the pre-clinical characterization, they will require rigorous product characterization to establish the safety and attenuation of virulence. Thus, it would be necessary to establish reproducible biomarkers of parasite characters such as non-virulence in manufactured lots of vaccines. Most of these attenuated strains of *L*. *donovani/infantum* have been produced by homologous recombination methods. More recently other methods to perform genome wide manipulation such as CRISPR-Cas and dimerised Cre recombinase based methods have also been optimized in *Leishmania* parasites^12, 13^.

In studies involving live attenuated parasites, the widely accepted standard is to produce a gene add back version of the null mutant parasite, most commonly in the form of an episomal plasmid vector expressing the gene of interest. Restoration of virulence under such conditions is considered adequate evidence for the specificity of attenuation. The ability of *Leishmania* parasites to rearrange their genomes upon experimental disruption of genes considered to be essential has been reported previously^14–17^. These rearrangements often involved duplication of certain fragments and/or altered expression of the genes as a compensatory effect^14, 18^. This raises important implications for characterization of the genetic stability of the null mutants as a function of attenuation of virulence and safety characteristics. Of the several gene deleted *Leishmania* parasites that are tested as potential vaccine candidates, none have undergone rigorous product characterization suggesting the desirability of developing more robust methods for this purpose.

Recent advancements in sequencing technologies and the wide availability of tools for bioinformatics analysis allowed whole genome sequencing methods to study molecular characteristics of various viral vaccines^19, 20^. Similar studies are lacking for parasitic vaccines. To obtain complete characteristics of attenuation, we have performed whole genome sequencing of *LdCen* ^−/−^ parasites and to our knowledge this is the first time whole genome sequence of a genetically modified parasite vaccine has been determined. Our results identify important characteristics of *LdCen* ^−/−^ parasites including additional deletions and altered expression of certain genes. However such alterations did not impact the attenuation characteristics of *LdCen* ^−/−^ caused by the deletion of the centrin gene. Together, our results demonstrate the utility of the whole genome sequencing method in obtaining reproducible and novel product characteristics that can be routinely used to assess the manufactured lots.

## Results

### Complete genome sequencing of *LdWT* and *LdCen*^−/−^ parasites

**Figure 1:**
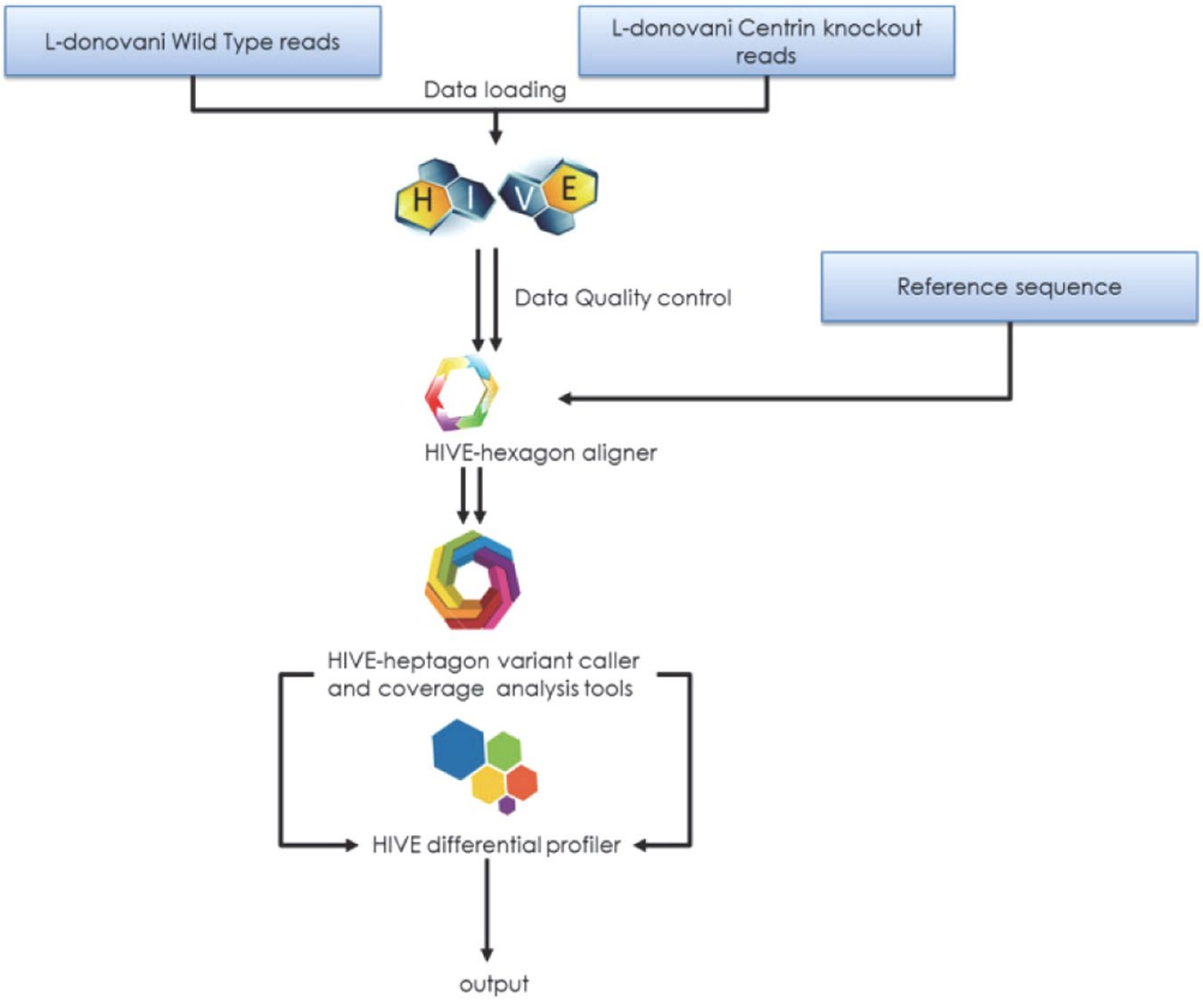
Bioinformatics workflow cataloging the steps taken to analyze the *LdWT* and *LdCen* ^−/−^ reads. These steps include (i) the sequencing of the two genomes, (ii) upload of the raw data into the HIVE system, (iii) validation through NGS quality controls, (iv) aligning the two sets of raw data independently against the reference genome (*L*. *infantum* or *L*. *donovani*) using the HIVE Hexagon aligner, (v) variant calls on the alignments using the HIVE Heptagon variant caller, (vi) mapping of the generated profiles (SNV calls and gaps) to determine differences between profiles (gaps of ≥300 bp), and (vii) manual validation of the results.

**Figure 2:**
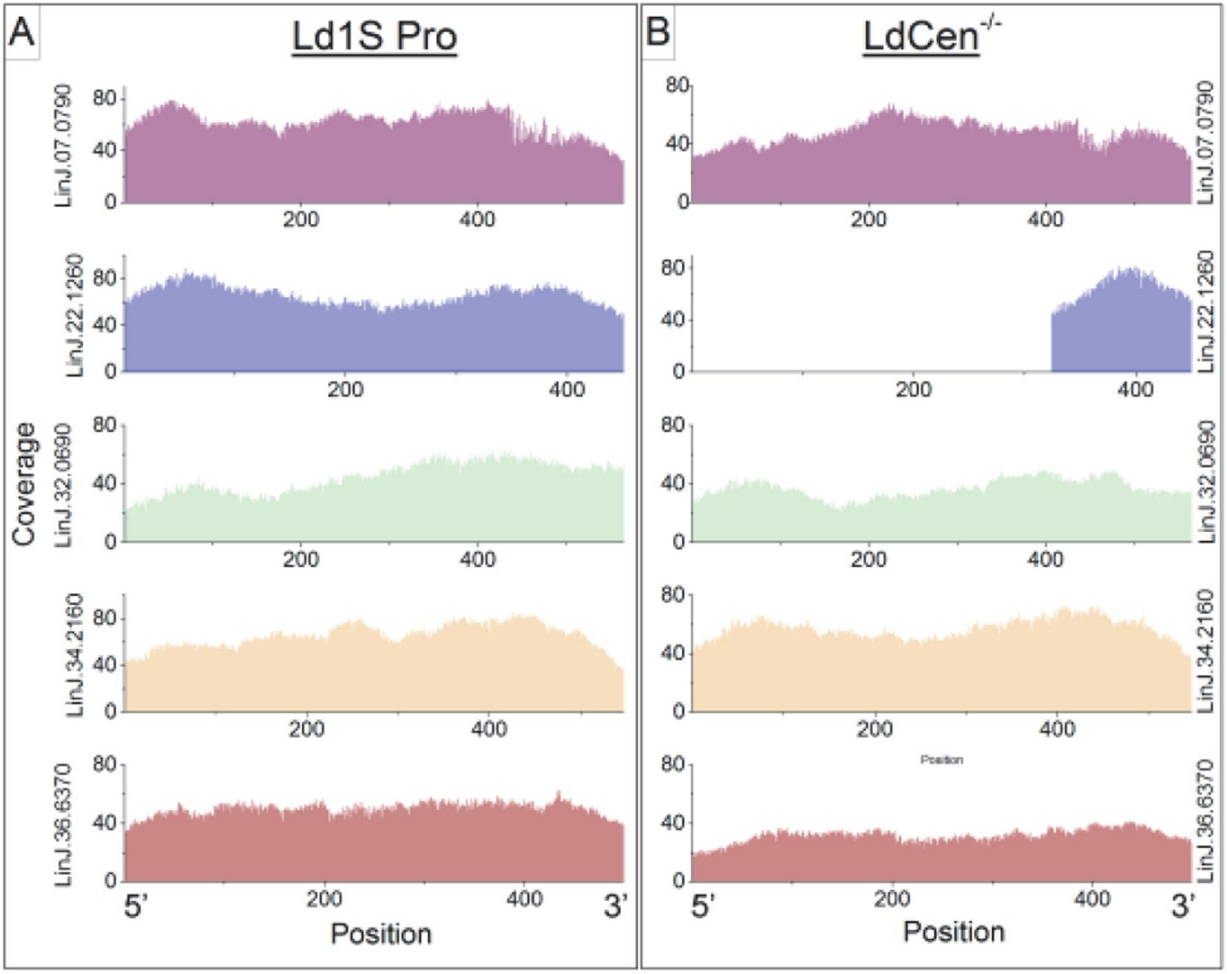
Sequence coverage of various Centrin homologs in *LdWT* and *LdCen* ^−/−^ genomes. Coverage maps of *LdWT* (**A**) and *LdCen* ^−/−^ (**B**) reads aligned against various Centrin homologs located on LinJ.07, LinJ.22, LinJ.32, LinJ.34, and LinJ.36 chromosomes. (**A**) All five centrin genes are covered in *LdWT*. (**B**) The centrin gene located on chromosome 22 (LinJ.22) is not covered, as expected, in the sequencing data from the *LdCen* ^−/−^ mutant. All other centrin genes are covered in the sequence data in *LdCen* ^−/−^.

Both the *LdWT* and *LdCen* ^−/−^ genomes were sequenced completely. The raw data from the sequencing reads were examined using analytical tools in the HIVE system. The alignments of the two sets of raw data were performed independently initially against the reference genome of *L*. *infantum* available on the genome database (www.tritrypdb.org) using the HIVE Hexagon aligner (Fig. 1). In addition, we have also used *Leishmania donovani* wild type genome as a reference (provided by Dr. Peter Myler, Genbank accession number to be provided) in situations where *L*. *infantum* genome was found inadequate for our analysis. The whole genome sequencing followed by coverage analysis of *LdWT*(Ld1S-2D) and *LdCen* ^−/−^ parasites revealed that the open reading frame corresponding to the centrin gene is deleted in the *LdCen* ^−/−^ genome as shown in previous studies (Fig. 2). The centrin locus in *LdCen* ^−/−^ was found to be replaced by hygromycin and neomycin resistance genes. The 325 bp of the centrin ORF from the 5′ end was found to be deleted as indicated by the lack of sequence coverage, and with only a ~125 bp sequence at the 3′ end left intact, identical to the results reported previously^21^ (Fig. 2B, LinJ.22.1260 panel). This partial deletion of *LdCen* ^−/−^ was by design since part of the ORF was used in assembling the targeting construct along with 3′UTR as shown in our previous studies. To confirm that the homologous recombination method used to delete centrin (LinJ.22.1260) did not perturb other centrin genes, we examined the other four centrin genes located on chromosomes 7, 32, 34 and 36 (LinJ.07.0790, LinJ.32.0690, LinJ.34.2160 and LinJ.36.6370). Results showed that none of the other four centrin genes are affected in the *LdCen* ^−/−^ mutant as indicated by the robust sequence coverage in the four loci in the *LdWT* and *LdCen* ^−/−^ genomes (Fig. 2).

### Comparison of *LdWT* and *LdCen*^−/−^ reveals additional deletions in multiple chromosomes in *LdCen*^−/−^

**Figure 3:**
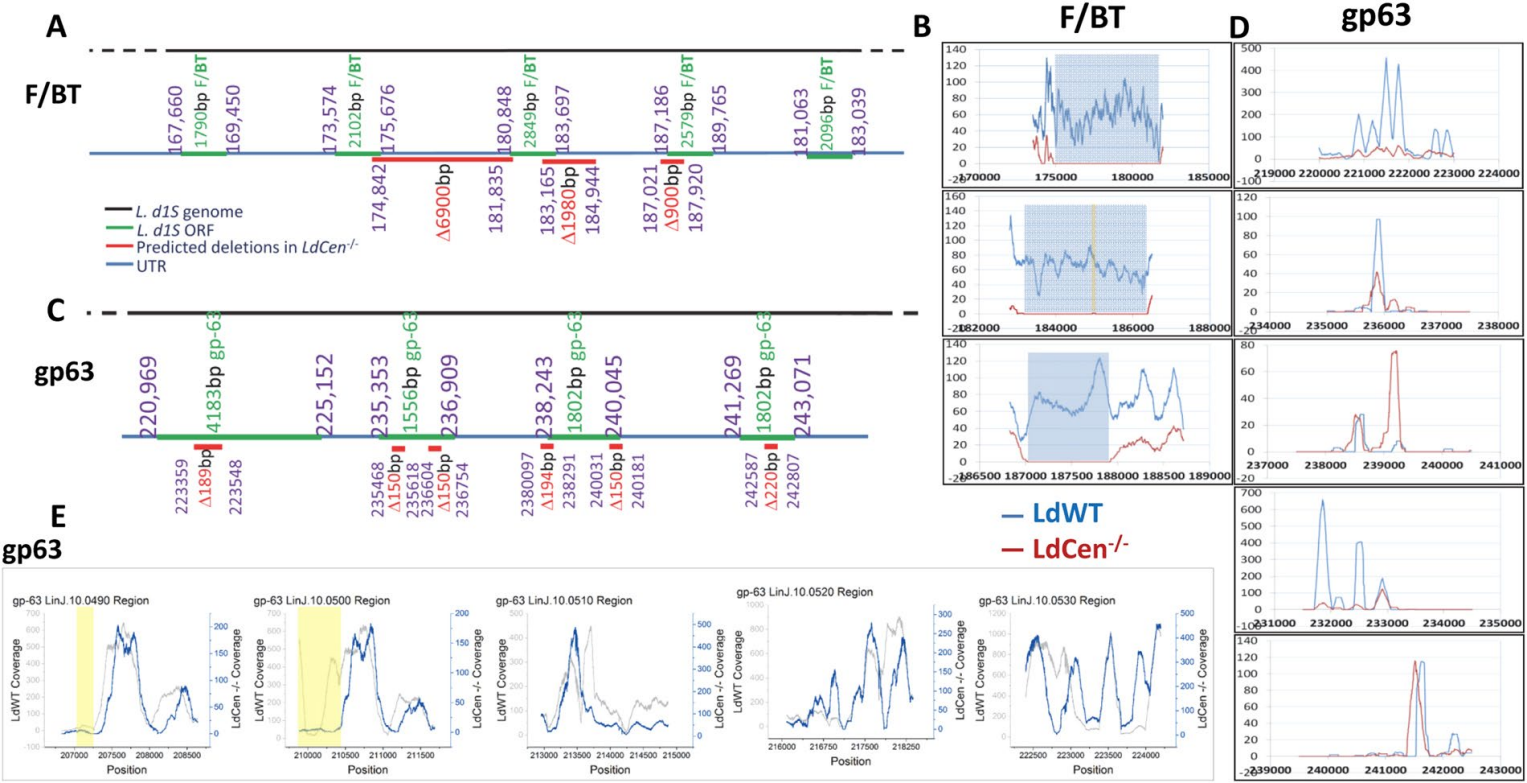
Additional deletions observed in *LdCen* ^−/−^ mutant in Chromosomes 10. (**A**) The deletions predicted by the bioinformatics analysis are shown as red bars. The gene arrangement in the reference *L*. *donovani* genome (as yet fully annotated) is shown as a black bar. The open reading frames are shown as green bars and the untranslated regions (UTR) are shown as blue bars. The size of the deletion is also indicated. Five copies of folate/biopterin transporter and gp63 genes are shown. The sixth folate/biopterin transporter gene located upstream of the five copies on chromosome 10 is not shown (corresponding to LinJ.10.0360). (**B**) Sequence coverage maps for the three tandem folate/biopterin genes are shown. (**C**) The deletions predicted in the gp63 region are shown as red bars. The gene arrangement in the reference *L*. *infantum* genome is shown as a black bar. (**D**) Sequence coverage of five gp63 genes on chromosome 10 is shown. (**E**) Sequence coverage of gp63 ORFs based on L infantum genome is shown. The yellow highlighted regions represent lack of coverage in *LdCen* ^−/−^ genome. The location identifiers for each of the genes are shown.

Results from genome-wide sequence comparison revealed additional deletions in several chromosomes in *LdCen* ^−/−^ compared to the parent strain *LdWT*. For the purpose of identifying bonafide deletions, we have used the following cut-off criteria. A sequence match of ≥120 nucleotides in both *LdWT* genome and the reference genome of *L*. *infantum* (www.tritrypdb.org) or *L*. *donovani*, and a stretch of sequence coverage of at least 300 nucleotides in order to be qualified as a deletion. This comparison would also preclude misidentification of deletions due to rearrangement of fragments between *LdWT*, *LdCen* ^−/−^ and *L*. *infantum*. Results showed that additional deletions occurred in *LdCen* ^−/−^ genome in several chromosomes (8, 10, 16, and 33) with the largest segments of deletions in chromosomes 10 and 16. The deletions in chromosome 10 largely occurred in a 40 kb region that contained tandem arrays of folate/Biopterin transporter and gp63 genes (Fig. 3A). In the folate/Biopterin tandem array, three large deletions ranging from 900 bp to as large as 6900 bp were observed in *LdCen* ^−/−^ genome in comparison with *L*. *donovani* reference genome. A complete lack of sequence coverage for the largest contiguous deletions is shown (Fig. 3B). A short segment of coverage of <100 bp was observed in the 6900 bp deletion (indicated as the yellow bar, Fig. 3B middle panel). Notably, our analysis of folate/Biopterin region using *L*. *infantum* as reference genome produced significantly different results in that instead of three large deletions (900 bp–6900 bp), multiple (15) short deletions (~200 to ~2200 bp) were observed (Supplementary Fig. 1). This suggested that the assembly of *L*. *infantum* genome might not be accurate in this region. Only deletions >250 are shown in the schematic diagrams since the average fragment used in nucleotide sequencing reaction was of 350 bp. A complete list of predicted deletions is provided in the supplementary information based on *L*. *donovani* and *L*. *infantum* reference genomes (Tables 1–10, Supplementary Information).

Additional deletions predicted on chromosome 10 corresponded to sequences coding for gp63 genes. The alignment parameters for HIVE pipeline are chosen such that it will not map >15% divergent reads to a reference map. Despite these constrained parameters, our analysis showed multiple deletions in the gp63 region of *LdCen* ^−/−^ compared to *L*. *donovani* reference genome (Fig. 3C). However, analysis of sequence coverage maps for the gp63 genes on chromosome 10 failed to identify clear lack of coverage in the gp63 region in *LdCen* ^−/−^ (Fig. 3D) as was observed in F/BT genes (Fig. 3B). To resolve this inconclusive result, we performed a comparative analysis using *L*. *infantum* as reference genome. Interestingly, this resulted in the identification of two deletions (Fig. 3E). This also suggested that the necessity of using multiple reference genomes in performing similar analysis to reliably ascertain the potential changes across the target regions.

We considered the possibility that the deletions could have been predicted due to ambiguous mapping between homologous regions where a single deletion is mis-mapped to multiple regions due to sequence similarity. However our analysis revealed that the deletions predicted in chromosome 10 comprising of sequences coding for three folate/biopterin transporter (F/BT) genes (Fig. 3A) were more homologous to each other than those without predicted deletions, indicating the robustness of our prediction. Hence the reads obtained from a covered region involving LinJ.10.0390, LinJ.10.0400, LinJ.10.0410 could not have mapped onto LinJ.10.0420 and LinJ.10.0380 by a chance of homology. Additionally the local dissimilarities within regions of these F/BT genes are larger than overall homology pointed in the table (Supplementary Fig. 2), thus ruling out inaccurate mapping by our algorithm. Similar reasoning also applies for gp63 genes that show significant local divergence despite a high overall degree of homology. The complete analysis of our results is available for review at the following web location https://hive.biochemistry.gwu.edu/dna.cgi?cmd=home&folder=/HIVE%20Space/Leishmania_publication/May2017.

### Experimental validation of the predicted deletions in *LdCen*^−/−^

**Figure 4:**
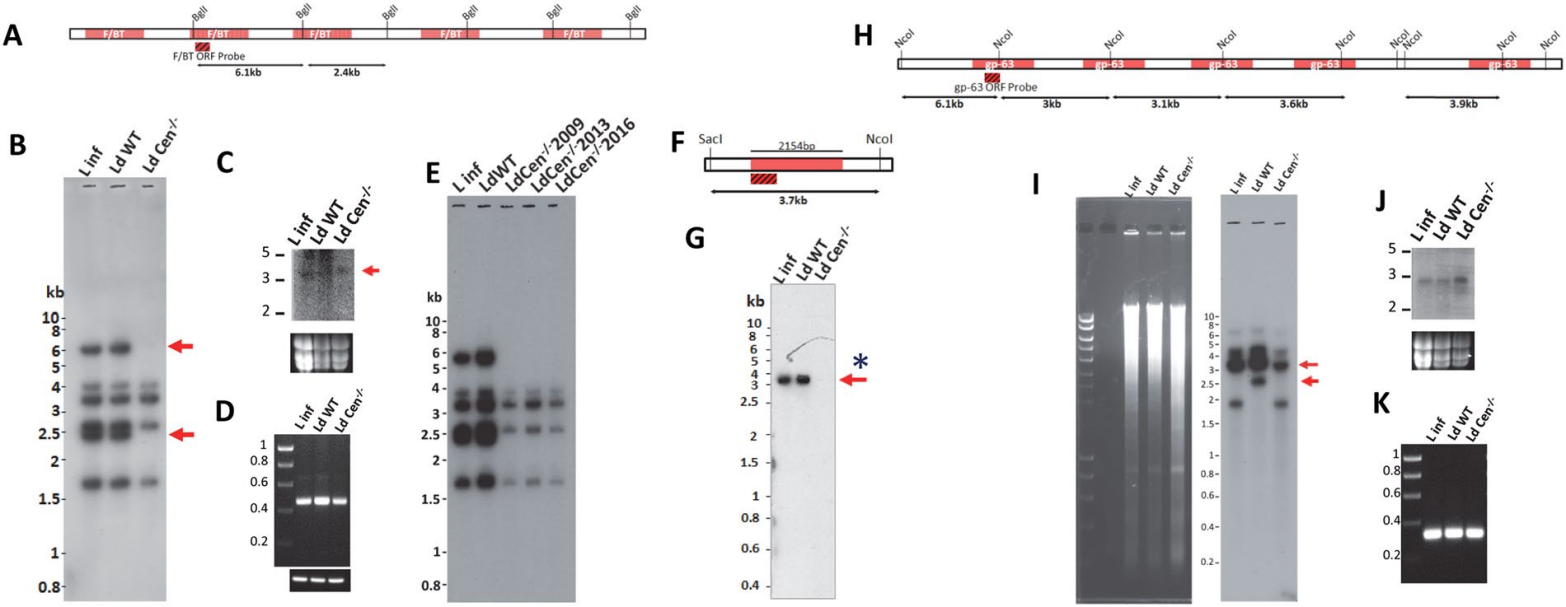
Experimental validation of the predicted deletions in the *LdCen* ^−/−^ genome. (**A**) The predicted sizes of the BglI digested fragments upon Southern hybridization and the probe used are indicated. (**B**) Southern hybridization of BglI digested *L*. *infantum*, *LdWT* and *LdCen* ^−/−^ gDNA is shown. (**C**) Northern blot with 20 µg of total RNA from *L*. *infantum*, *LdWT* and *LdCen* ^−/−^ parasites hybridized with the folate/biopterin transporter probe is shown. The ribosomal RNA bands as loading controls are shown at the bottom. (**D**) RT-PCR with 1 µl product of the first strand cDNA synthesis reaction that amplified a fragment of the folate/biopterin transporter ORF. Loading control showing a comparable amplification of an unrelated gene fragment (LdUfsp1) is at the bottom. (**E**) Southern blot hybridization of folate/biopterin transporter genes with three additional stocks of LdCen^−/−^ parasites collected over a period of 7 years (2009, 2013 and 2016). (**F**) The predicted size of SacI-NcoI digestion fragment and the probe corresponding to Δ2154 bp deletion in *LdCen* ^−/−^ genome are shown. (**G**) Southern hybridization with a ^32^p labeled probe derived from the 2154 bp deletion upon SacI-NcoI digested *L*. *infantum*, *LdWT* and *LdCen* ^−/−^ gDNA is shown. Absence of the predicted 3.7 kb fragment in *LdCen* ^−/−^, is indicated with a red arrow. (**H**) The predicted restriction map upon NcoI digestion of genomic DNA for tandem gp63 copies is shown. The location of the probe used in hybridization experiments is indicated with red. The predicted sizes of the fragments upon Southern hybridization are indicated as black arrows. (**I**) Agarose gel showing the NcoI digested *L*. *infantum*, *LdWT* and *LdCen* ^−/−^ gDNA. Southern hybridization of NcoI digested *L*. *infantum*, *LdWT* and *LdCen* ^−/−^ gDNA is shown. Reduced intensity of a ~3 kb fragment in *LdCen* ^−/−^ lane compared to *L*. *infantum* and *LdWT* lanes is indicated with a red arrow. (**J**) Northern blot with 20 µ g of total RNA from *L*. *infantum*, *LdWT* and *LdCen* ^−/−^ parasites upon hybridization with the gp63 probe is shown. The ribosomal RNA bands as loading controls are shown at the bottom. (**K**) RT-PCR with 1 µl product of the first strand cDNA synthesis reaction that amplified a fragment of the gp63 ORF.

To verify if the deletions shown in the sequence comparison studies using bioinformatics tools above are accurate, we performed Southern blot analysis of genomic DNA. For this purpose, we have selected a subset of deletions that correspond to protein coding regions and in some cases, untranslated regions. Our analysis showed that deletions in chromosome 10 concentrated in both the coding and untranslated regions corresponding to folate/biopterin transporter gene alleles as well as gp63 gene alleles. To verify the loss of several genomic DNA fragments in the folate/biopterin transporter and gp63 gene alleles, we first performed Southern hybridization experiments. Both the reference *L*. *infantum* and the parent *LdWT* genomes contain five copies of folate/biopterin transporter genes that are arranged in tandem (Fig. 3A) with an additional sixth copy upstream of the tandem cluster. The nucleotide sequence similarities between five contiguous copies of the folate/biopterin transporter genes range from 70–98% as calculated using multiple sequence alignment tool MUSCLE (http://www.ebi.ac.uk/Tools/msa/muscle, Supplementary Fig. 2A). As the schematic diagram shows, digestion with the restriction enzyme BglI would result in 6 DNA fragments detectable upon hybridization with a ^32^p labeled probe derived from the folate/biopterin transporter coding sequence (Fig. 4A). The DNA hybridization results showed loss of DNA fragments (corresponding to 6.1 Kb and 2.4 Kb) in *LdCen* ^−/−^ but not in *LdWT* and *L*. *infantum* genomes (Fig. 4B, red arrows). These experimental results are consistent with the deletions predicted by our analysis using *L*. *donovani* reference genome (Fig. 3A). To further confirm if loss of the folate/biopterin transporter gene fragments affected transcription, we performed Northern blot hybridization with total RNA obtained from the promastigote stage of the three parasites strains. As the results showed, mRNA corresponding to the folate/biopterin transporter was detectable from all three parasites tested viz., *L*. *infantum*, *LdWT* and *LdCen* ^−/−^ (Fig. 4C). Ribosomal RNA bands from the formaldehyde gels showed a comparable amount of total RNA in the lanes between *LdWT* and *LdCen* ^−/−^ parasites. To further confirm the extent of transcription, we performed RT-PCR with the same RNA preparations used in the Northern blot hybridization. Results showed folate/biopterin transporter transcription occurred in all three parasites tested, consistent with the Northern blot results (Fig. 4D). To further confirm whether the deletions in folate/biopterin transporter genes were stable over time and could be reliably used as biomarkers, we selected two additional stocks of *LdCen* ^−/−^ parasites that have undergone extensive cycling through laboratory animal infections and *in vitro* culture over a period of 7 years and performed Southern blot hybridization as described above (Fig. 4B). The results showed that the three different stocks of *LdCen* ^−/−^ contained identical deletions (absence of 6.1 Kb and 2.4 Kb fragments). The observation that the deletions were maintained stably over a period of 7 years suggests that the additional deletions could have arisen during the initial process of targeted Centrin deletion by homologous recombination in *L*. *donovani* (Fig. 4E).

To further verify other deletions, we selected to experimentally demonstrate the loss of the 2154 bp fragment located in the untranslated region between the third and fourth copies of the folate/biopterin transporter gene alleles. A SacI-NcoI digestion would result in a 3.7 kb DNA fragment detectable with a 500 bp ^32^p labeled probe derived from the 5′ end of the predicted deletion (Fig. 4F). The DNA hybridization results showed a clear loss of the 2154 bp fragment in the *LdCen* ^−/−^ genome but not in the *LdWT* and *L*. *infantum* genomes (Fig. 4G).

Next we analyzed the deletions occurred in two of the 5 tandem copies of gp63 gene. Since gp63 is a known virulence factor and extensively studied for its role in *Leishmania* pathogenesis, we sought to verify the deletion of DNA fragments in gp63 genes in the *LdCen* ^−/−^ genome. The gp63 gene family consists of homologous copies arranged in tandem (Fig. 3C). The deletions (250 bp and 740 bp) in the *LdCen* ^−/−^ genome correspond to the coding regions in the first and second copies of the gp63 genes. The gp63 genes share a high degree of homology ranging from 87 to almost 100% (Supplementary Fig. 2B), with the gp63 in *L*. *infantum* (LinJ.10.0520) that contains an unusual 492 bp extension at the 5′end that is not found in the rest of the members. To verify the deletions in gp63, we have performed a Southern hybridization with NcoI digested gDNA from *L*. *infantum*, *LdWT* and *LdCen* ^−/−^. NcoI digestion followed by hybridization with a 350 bp ^32^p labeled probe derived from the coding region of gp63 containing identical nucleotide sequence shared between all five copies of gp63 would yield 5 distinct bands (Fig. 4H). Results showed that, although the deletions were not as clear cut as those observed in the F/BT region, *LdCen* ^−/−^ indeed lost copies of gp63 coding regions as indicated by the reduced band intensity corresponding to ~3.5 kb on the Southern blot (Fig. 4I, Southern blot), although the DNA gel shows comparable amount of DNA in all the three lanes (Fig. 4I, ethidium bromide stained agarose gel). The restriction map shown in Fig. 4H is based on the reference *L*. *infantum* genome (www.tritrypdb.org). However the locations of NcoI sites in the *L*. *donovani* wild type parent strain used in the study are slightly different and the pattern of bands shown in Fig. 4I is consistent with that observation. *LdCen* ^−/−^ does not show the 2.6 kb band due to the additional deletions identified. Northern hybridization with the same ^32^p labeled gp63 probe showed that *LdCen* ^−/−^ contained similar level of gp63 transcripts compared to *L*. *infantum* and *LdWT* (Fig. 4J). Ribosomal RNA bands from the formaldehyde gels showed a comparable amount of total RNA in the lanes between *LdWT* and *LdCen* ^−/−^ parasites. To clearly demonstrate the extent of transcription, we performed RT-PCR with the same RNA preparations used in the Northern blot hybridization. Results showed that comparable level of gp63 transcription occurred in all three parasites tested, consistent with the Northern blot results (Fig. 4K).

**Figure 5:**
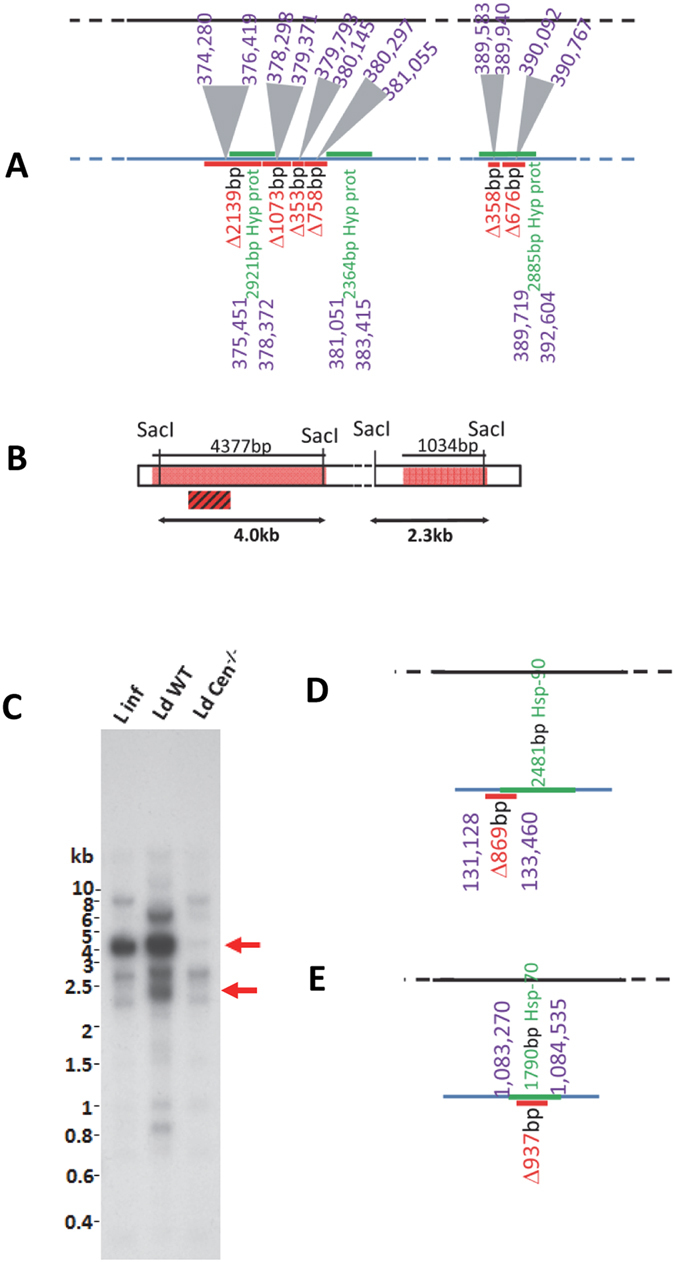
Additional deletions observed in *LdCen* ^−/−^ mutant in Chromosomes 16. (**A**) The deletions predicted by the bioinformatics analysis are shown as red bars. The gene arrangement in the reference *L*. *donovani* genome with the gene listing is shown as a black bar. The open reading frames are shown as green bars and the untranslated regions (UTR) are shown as blue bars. The size of the deletion is also indicated. The predicted hypothetical protein coding genes are indicated with the gene identifiers are shown. (**B**) The restriction map upon SacI digestion is shown. The location of the probe used in hybridization experiments is indicated with red. The predicted size of the fragment upon Southern hybridization is indicated as black arrows. (**C**) Southern hybridization of SacI digested *L*. *infantum*, *LdWT* and *LdCen* ^−/−^ gDNA is shown. Absence of the 4.0 kb and 2.3 kb bands is indicated with a red arrow. Additional deletions in *LdCen* ^−/−^ genome ranging from 700–900 bp found in chromosomes (**D**) 33 and (**E**) 8 and the nearest predicted protein coding ORFs are indicated.

To confirm the deletions predicted on other chromosomes, notably chromosome 16, where several large deletions occurred in a 15 kb locus out of 689 Kbp, (total size of chromosome 16), we selected to detect the 4377 bp fragment. Notably, the 4377 bp deletion comprised of four contiguous deletions (2139 bp, 1073 bp, 353 bp and 758 bp, Fig. 5A) separated by short sequence coverages amounting to <30 bp. These coverages could be due to repeat regions and for practical purposes could be considered as a single 4377 bp deletion. The 4377 bp fragment overlaps two regions encoding two hypothetical proteins containing Stealth Protein CR2 domains (LinJ.16.1030a and LinJ.16.1040, Fig. 5A). A SacI digestion would result in a 4 kb DNA fragment and a 2.3 kb fragment detectable with a 650 bp ^32^p labeled probe derived from the 5′ end of the predicted deletion (Fig. 5B). The DNA hybridization results showed a clear loss of the 4 kb and 2.3 kb fragments from in the *LdCen* ^−/−^ genome but not in the *LdWT* and *L*. *infantum* genomes (Fig. 5C). The 4377 bp region contained several repeat regions and expectedly, several fragments reacted with the 650 bp probe giving additional reaction products upon Southern hybridization (Fig. 5C). Additional deletions ranging from 800–900 bp are also found in chromosomes 33 and 8 that encode putative heat shock proteins (Fig. 5D,E respectively) based on our analysis using *L*. *donovani* reference genome. A complete list of predicted deletions is provided in the supplementary information based on *L*. *donovani* and *L*. *infantum* reference genomes (Tables 1–10, Supplementary Information).

## Discussion

The protective immunity induced by live attenuated *Leishmania* parasite vaccines is being recognized by the development of several distinct gene deletion mutants as potential vaccine candidates. Immunization with a *L*. *donovani/infantum* gene deletion mutants lacking biopterin transporter^22^ (BT1), Centrin^5^, p27^23^, Hsp70^24^ or ALO^11^, protected mice against virulent challenge. Similarly, *L*. *donovani* partial knockout parasites lacking A2-A2rel gene cluster^25^ and SIR2^26^ gene in *L*. *infantum* have also been shown to induce protection in experimental mouse infections. These genetic mutant parasites have been developed typically by homologous recombination methods. Since genetic alterations in several null mutants of *Leishmania* parasites have been reported previously^27–29^, identifying biomarkers of attenuation of virulence for the potential vaccine candidates is essential. A more robust characterization of the genetically modified live attenuated organisms would enable monitoring of the properties of attenuation of virulence reproducibly and potentially maintenance of immunogenicity when some of these experimental stage vaccines are manufactured at industrial scale for clinical use.

*Leishmania* parasites are auxotrophs for folates and obtain folates from the growth medium. Previous studies in *Leishmania* have shown that Biopterin transporters can also transport folate^27^. Biopterin has been known to promote the growth of *L*. *donovani* parasites in culture. However this growth promoting property was only evident in freshly isolated parasites. Once the parasite cultures are adapted to grow *in vitro*, the exogenous addition of Biopterin did not make any difference to the parasite growth^28^. Comparative gene expression analysis revealed an overexpression of folate/biopterin transporter transcripts and an increased transport activity in cultures adapted for *in vitro* growth. These results indicated that adaptation of cultures for *in vitro* growth could lead to altered expression/activity of folate/biopterin transporters. Since in the process of deleting centrin gene, we found that additional deletions occurred in two known genes in *LdCen* ^−/−^, folate/biopterin transported and gp63, we analyzed their expression in the attenuated strain.

In addition to the six copies of putative folate/biopterin transporter genes on chromosome 10, (LinJ.10.0360; LinJ.10.0380; LinJ.10.0390; LinJ.10.0400 LinJ.10.0410; LinJ.10.0420, Supplementary Fig. 1) several other copies of folate/biopterin transporter genes have been identified in the *L*. *infantum* genome (http://tritrypdb.org/tritrypdb/; LinJ.04.0020; LinJ.06.0310; LinJ.19.0870). Such organization of F/BT gene copies in the *L donovani* genome we used in our study as additional reference genome is yet to be ascertained. Of these copies, folate/biopterin transporter corresponding to LinJ.10.0420 has been experimentally shown to be a high affinity folate transporter (also known as FT5)^17^. The nucleotide sequence of the probe used in our hybridization studies can react with the four copies (LinJ.10.0380; LinJ.10.0390; LinJ.10.0400 LinJ.10.0410) due to high degree of sequence identity and to a lesser extent with LinJ.10.0420. However, our experimental results demonstrating the absence of 6.1 kb and 2.4 kb fragments on the Southern blot hybridization in *LdCen* ^−/−^, clearly demonstrate the deletions predicted in our bioinformatics analysis using *L*. *donovani* genome as reference are accurate. Similarly, both the Northern hybridization and RT-PCR results showing the expression of folate/biopterin transporter in *LdCen* ^−/−^ do not fully account for transcripts from the other copies of the gene located elsewhere due to sequence divergence. Therefore it is likely that the level of expression from folate/biopterin transporter genes does occur in *LdCen* ^−/−^ parasites to a similar degree to that of *LdWT* despite the loss of 6.1 and 2.4 kb fragments encompassing the F/BT coding regions. This is corroborated by our previously published results where expression of an episomal copy of centrin fully restored the growth of *LdCen* ^−/−^ mutants^21^. Studies in *L*. *donovani1S* strain that contained two alleles of BT-1 have shown that disruption of BT1 caused loss of virulence as indicated by reduced growth *in vitro* and *in vivo* of the BT-1 null mutants^22^. Similar growth defects were also observed in *L*. *major* BT1 null mutant^29^. Taken together, the unexpected loss of folate/biopterin transporter gene copies both in the genomic loci and their unaltered transcription in *LdCen* ^−/−^ is revealing. Our results showing that 3 of the 5 copies of folate/biopterin transporter genes are disrupted in *LdCen* ^−/−^ could provide novel biomarkers to verify the authenticity of the manufactured stocks.

Leishmania gp63 has been one of the most extensively studied virulence factors. The abundance of gp63 on the surface of *Leishmania* parasites, wide biochemical substrates for its activity, and its ability to alter the host signaling have been very well documented^30^. Loss of virulence was also reported due to deletion of gp63 in *L*. *major*, identifying gp63 as a virulence factor^31^. In our characterization of *LdCen* ^−/−^ parasites even though we found a few copies of gp63 were disrupted, it did not have any impact on its transcripts as indicated by both the Northern blot hybridization and RT-PCR analysis. Interestingly, we obtained distinctly different results when using *L*. *infantum* and *L donovani* as reference genomes. This suggests that the organization of gp63 genes could be different in these genomes. Further, there is a significant inhomogeneity in the coverage of the regions where gp63 repeats are located (see sample of such coverage on Fig. 3D). The reason for this phenomenon might be due to reference assembly artifacts if different alleles of polyploid genomes accumulate different recombination patterns. Our gap analysis software assumes a continuous coverage and therefore the interpretation of differential coverages in this region still remains somewhat unclear. However our Southern blot results clearly demonstrate the loss/differential arrangement of gp63 genes in the *LdCen* ^−/−^ genome possibly induced during the selection process either in both alleles or selectively in one allele. Resolution of this result requires further study.

Presence of gp63 mRNA in *LdCen* ^−/−^ indicated that the loss of virulence observed in *LdCen* ^−/−^ was due to the targeted deletion of centrin alone. Additionally, in *L*. *infantum* genome the gp63 tandem array on chromosome 10 contains five copies as indicated in Supplementary Fig. 1. *L*. *infantum* genome contains three additional gp63 genes on chromosomes 28 and 31. However, the sequence divergence between those and the tandem array located on chromosome 10 is far too high and the gp63 probe used in our study does not show any reactivity with LinJ.31.2040, LinJ.28.0600 and LinJ.28.0610 due to lack of sequence complementarity. Thus the deletions identified by us on chromosome 10 represent verifiably true and not due to misalignment. Finally, re-expression of centrin gene in *LdCen* ^−/−^ parasites restored the infectivity suggesting that loss of centrin is responsible for attenuation in virulence. Deletion of fragments corresponding to coding regions of gp63 in *LdCen* ^−/−^ parasites in the current study showed similar utility as biomarkers as was seen in the folate/biopterin transporters. Deletion of fragments in repeat regions containing Stealth proteins CR2 conserved domains (http://pfam.xfam.org/family/PF11380) and certain putative heat shock proteins remains to be investigated further.

The biological relevance of other deletions observed in other chromosomes, most frequently in untranslated regions is unknown at this time and will be investigated in future. More important questions such as whether deletions unrelated to centrin occur spontaneously or due to selection pressure using antibiotic markers such as Hygromycin and Neomycin remain to be studied. Previous studies reported that attempts to delete GSH1 gene have inadvertently caused loss of a downstream gene, LinJ.18.1670^18^ indicating that drug selection pressure could cause such changes. To partly address the origin of the additional deletions identified in *LdCen* ^−/−^, we analyzed two additional stocks of *LdCen* ^−/−^ parasites that have undergone extensive cycling through laboratory animals and *in vitro* culture over a period of 7 years. Southern blot hybridization revealed that the deletions in the F/BT region are stably maintained over this prolonged period, suggesting that the deletions most likely occurred during our initial attempts to delete centrin by homologous recombination. Stability of the additional deletions along with centrin gene deletion identified by us underscores their value as biomarkers. Thus our study highlights the practical utility of these novel biomarkers of attenuation in characterization of clinical lots of *LdCen* ^−/−^ parasites in conjunction with previously demonstrated characteristics of *LdCen* ^−/−^ parasites. Therefore, the whole genome sequencing method to characterize *LdCen* ^−/−^ parasites demonstrates the desirability of such unbiased characterization and practical utility in providing novel biomarkers of attenuation in parasitic vaccine candidates.

## Methods

### Parasites

*Leishmania donovani* promastigotes were grown in M199 medium containing 10% heat inactivated fetal bovine serum. Clones of both wild type parent strain (Ld1S2D) and the centrin deletion mutant (*LdCen* ^−/−^) from the stocks stored in liquid nitrogen and not maintained in prolonged culture were selected on Nobel agar plates. The *LdCen* ^−/−^ clones were selected from the plates containing hygromycin (80 µg/ml) and neomycin (100 µg/ml). Two clones from each strain of parasites were used for genomic DNA isolation using Promega genomic DNA Wizard kit.

### Complete genome sequencing

Complete genome sequencing of two clones each from *LdWT* and *LdCen* ^−/−^ was determined by MiSeq genome sequencing reaction on an Illumina sequencing instrument at the sequencing core facility at the Center for Biologics Evaluation and Research.

### Comparison of *L*. *infantum*, *LdWT* and *LdCen*^−/−^ genomes

*LdCen* ^−/−^ and LdWT paired end sequence reads were uploaded into the HIVE system and quality control procedures were executed^32–34^ for the reads in order to detect potential issues with sequencing and ensure high quality of the short sequences. Additionally, HIVE-censuscope^35^ was used to screen the sequences, identify taxonomical identity and the nearest reference neighbors. Both sets of reads were aligned against *Leishmania infantum JPCM5* reference genome (retrieved from www.tritrypdb.org) using HIVE-hexagon short read alignment algorithm^36^. In certain cases, we have also used *L*. *donovani* genome as reference (provided by Dr. Peter Myler) Minimum alignment match length was chosen (120 nucleotides match) in a manner to consider only non-partial, high fidelity alignments. All other parameters were maintained as recommended defaults useful for analyzing eukaryotic parasitic genomes of this size. Next, the HIVE Heptagon variant caller^37^ was used to generate perform sequence pileup, vertical and horizontal converge analysis, detect SNVs, insertion and deletions, gaps and contigs for each of the sample alignments. Gap information generated by the profiler was used with the HIVE Annotation Mapper software to compare the alignments of the *LdWT* and *LdCen* ^−/−^. Regions of the reference genome that were covered with statistically significant coverage in one of the samples, but not in the other, and of at least 300 bp size, were reported.

### Southern/Northern hybridization

Total genomic DNA was isolated from promastigotes with the Wizard genomic DNA purification kit (Promega Biosciences). Total RNA was isolated from the promastigotes using Pure Link mini RNA isolation kit (Ambion). The DNA was digested with restriction enzymes with either SacI alone or SacI-NcoI and the digestion products were separated on 1% agarose gels and transferred to positively charged nitrocellulose membranes. Southern blot analysis of the resolved DNA was performed as described previously using a ^32^p-labelled *L*. *donovani* partial sequence derived from Δ2154 bp and Δ1854 bp deletions as probes^38^. The DNA fragments, used as probes on the Southern hybridization, were PCR amplified with primers located in the regions representing the Δ2154 bp and Δ1854 bp deletions. These DNA fragments were ligated into pCR2.1-Topo vector and the nucleotide sequence of the probes was determined to ensure fidelity. The plasmid containing the correct probe was digested with EcoRI, gel purified and labeled with Random Prime it-II kit using ^32^p-dCTP (Agilent Technologies). Total RNA (20 μg/lane) prepared from mid-log phase promastigote cultures was resolved on a formaldehyde gel and blotted onto a nitrocellulose membrane. To verify the transcription of mRNA encoding biopterin transporter in *LdCen* ^−/−^ parasites, a 480 bp DNA fragment (from the putative Folate/biopterin transporter open reading frames) or a 350 bp gp63 DNA fragment from its open reading frame were used as a probe in Northern blot hybridization.

### RT-PCR

Total RNA isolated from *Leishmania* parasites was treated with RNase free DNase (PureLink RNA mini kit, Ambion). First strand cDNA synthesis was performed with 500 ng of DNase treated RNA (SuperScript first strand synthesis, Thermofisher) followed by RNaseH treatment. PCR performed with the resulting cDNA using primers corresponding to folate/biopterin transporter or gp63 genes. Only a partial open reading frame is amplified in the PCR.

## Electronic supplementary material

Supplementary information
