# Supplementary material for: Whole genome sequencing of live attenuated Leishmania donovani parasites reveals novel biomarkers of attenuation and enables product characterization
Source: Sci Rep. 2017 Jul 5;7:4718. doi: 10.1038/s41598-017-05088-4 (PMC5498541; doi:10.1038/s41598-017-05088-4)
Supplement: Supplementary file 1 — Supplementary information [file 41598_2017_5088_MOESM1_ESM.doc]

**Whole genome sequencing of live attenuated *Leishmania donovani* parasites reveals novel biomarkers of attenuation and enables product characterization**

Sreenivas Gannavaram^1^, John Torcivia^2^, Lusine Gasparyan^3^, Amit Kaul^1^, Nevien Ismail^1^, Vahan Simonyan^2^, Hira L. Nakhasi^1^


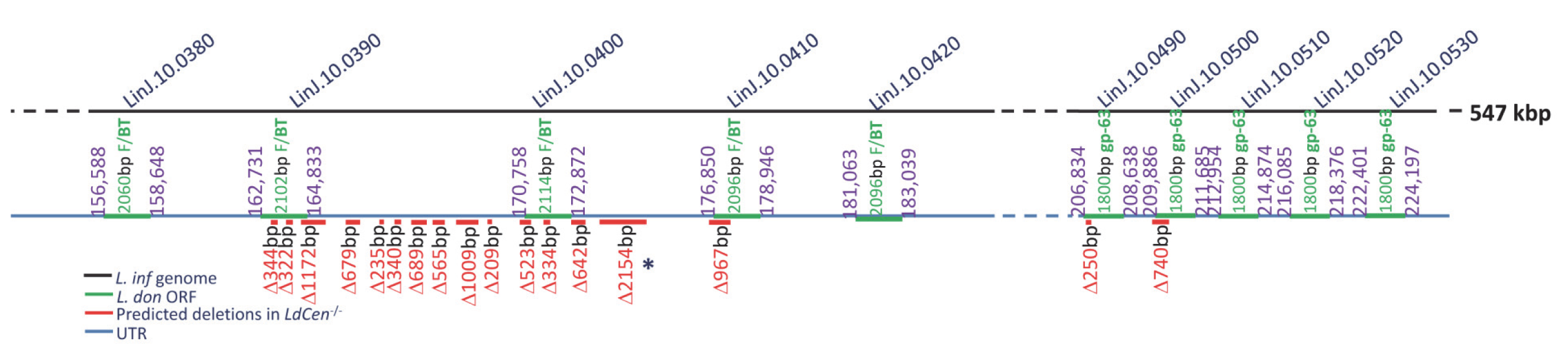


Supplementary Figure 1: The chromosome 10 region corresponding to F/BT and gp63 regions based on *L. infantum* reference genome is shown. The region between the folate/biopterin transporter and gp63 clusters (~5kb) is not shown (dotted lines). The asterisk corresponds to the 2154bp deletion.


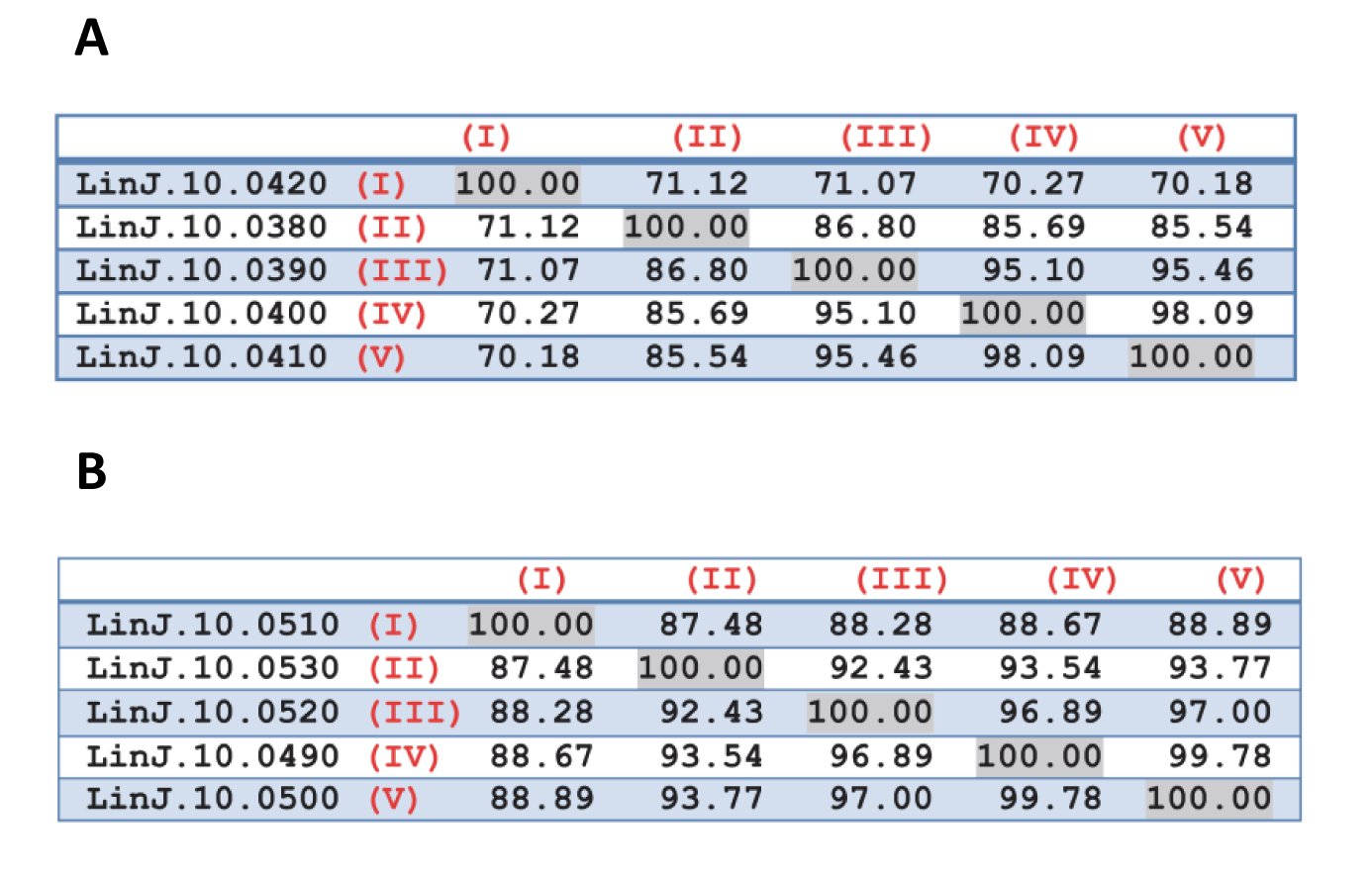


Supplementary Figure 2: Sequence homology of F/BT and gp63 genes based on *L. infantum* genome. A) Nucleotide sequence homology between the 5 homologs of folate/biopterin transporter and, B) five homologs of gp63 genes on chromosome 10 is shown.

**Supplementary information**

**Table I: Deletions in *LdCen*^-/-^ genome chromosome #10 compared to *L. donovani*** genome

| **Reference genome** | **Start** | **End** | **Size of deletion (bp)** |
| --- | --- | --- | --- |
| **LdoS_10 | Leishmania donovani | 1 to 600849** | 174842 | 181835 | 6994 |
| **LdoS_10 | Leishmania donovani | 1 to 600849** | 183165 | 184944 | 1780 |
| **LdoS_10 | Leishmania donovani | 1 to 600849** | 185031 | 186385 | 1355 |
| **LdoS_10 | Leishmania donovani | 1 to 600849** | 187021 | 187920 | 900 |
| **LdoS_10 | Leishmania donovani | 1 to 600849** | 242587 | 242807 | 221 |
| **LdoS_10 | Leishmania donovani | 1 to 600849** | 238097 | 238291 | 195 |
| **LdoS_10 | Leishmania donovani | 1 to 600849** | 174083 | 174260 | 178 |
| **LdoS_10 | Leishmania donovani | 1 to 600849** | 235468 | 235618 | 151 |
| **LdoS_10 | Leishmania donovani | 1 to 600849** | 236604 | 236754 | 151 |
| **LdoS_10 | Leishmania donovani | 1 to 600849** | 240031 | 240181 | 151 |
| **LdoS_10 | Leishmania donovani | 1 to 600849** | 223359 | 223508 | 150 |
| **LdoS_10 | Leishmania donovani | 1 to 600849** | 234992 | 235134 | 143 |
| **LdoS_10 | Leishmania donovani | 1 to 600849** | 243511 | 243652 | 142 |
| **LdoS_10 | Leishmania donovani | 1 to 600849** | 230398 | 230527 | 130 |
| **LdoS_10 | Leishmania donovani | 1 to 600849** | 168218 | 168340 | 123 |

**Table 2: Deletions in *LdCen*^-/-^ genome chromosome #16 compared to *L. donovani* genome**

| **Reference genome** | **Start** | **End** | **Size of deletion (bp)** | |
| --- | --- | --- | --- | --- |
| **LdoS_16 | Leishmania donovani | 1 to 706471** | 385032 | 387903 | 2872 |  |
| **LdoS_16 | Leishmania donovani | 1 to 706471** | 374280 | 376419 | 2140 |  |
| **LdoS_16 | Leishmania donovani | 1 to 706471** | 383344 | 384671 | 1328 |  |
| **LdoS_16 | Leishmania donovani | 1 to 706471** | 388203 | 389431 | 1229 |  |
| **LdoS_16 | Leishmania donovani | 1 to 706471** | 378298 | 379371 | 1074 |  |
| **LdoS_16 | Leishmania donovani | 1 to 706471** | 380297 | 381055 | 759 |  |
| **LdoS_16 | Leishmania donovani | 1 to 706471** | 390092 | 390767 | 676 |  |
| **LdoS_16 | Leishmania donovani | 1 to 706471** | 389583 | 389940 | 358 |  |
| **LdoS_16 | Leishmania donovani | 1 to 706471** | 379793 | 380145 | 353 |  |
| **LdoS_16 | Leishmania donovani | 1 to 706471** | 577698 | 577856 | 159 |  |
| **LdoS_16 | Leishmania donovani | 1 to 706471** | 580702 | 580852 | 151 |  |
| **LdoS_16 | Leishmania donovani | 1 to 706471** | 576980 | 577123 | 144 |  |
| **LdoS_16 | Leishmania donovani | 1 to 706471** | 580540 | 580680 | 141 |  |
| **LdoS_16 | Leishmania donovani | 1 to 706471** | 365571 | 365690 | 120 |  |
| **LdoS_16 | Leishmania donovani | 1 to 706471** | 379523 | 379641 | 119 |  |
| **LdoS_16 | Leishmania donovani | 1 to 706471** | 578166 | 578281 | 116 |  |
| **LdoS_16 | Leishmania donovani | 1 to 706471** | 372608 | 372710 | 103 |  |

**Table 3: Deletions in *LdCen*^-/-^ genome chromosome #8 compared to *L. donovani* genome**

| **Reference genome** | **Start** | **End** | **Size of deletion (bp)** |
| --- | --- | --- | --- |
| **LdoS_08 | Leishmania donovani | 1 to 566295** | 299133 | 299361 | 229 |
| **LdoS_08 | Leishmania donovani | 1 to 566295** | 523026 | 523232 | 207 |
| **LdoS_08 | Leishmania donovani | 1 to 566295** | 310556 | 310753 | 198 |
| **LdoS_08 | Leishmania donovani | 1 to 566295** | 315309 | 315505 | 197 |
| **LdoS_08 | Leishmania donovani | 1 to 566295** | 314356 | 314532 | 177 |
| **LdoS_08 | Leishmania donovani | 1 to 566295** | 309126 | 309296 | 171 |
| **LdoS_08 | Leishmania donovani | 1 to 566295** | 313450 | 313619 | 170 |
| **LdoS_08 | Leishmania donovani | 1 to 566295** | 527200 | 527368 | 169 |
| **LdoS_08 | Leishmania donovani | 1 to 566295** | 299897 | 300047 | 151 |
| **LdoS_08 | Leishmania donovani | 1 to 566295** | 303639 | 303789 | 151 |
| **LdoS_08 | Leishmania donovani | 1 to 566295** | 304626 | 304776 | 151 |
| **LdoS_08 | Leishmania donovani | 1 to 566295** | 442263 | 442413 | 151 |
| **LdoS_08 | Leishmania donovani | 1 to 566295** | 300247 | 300396 | 150 |
| **LdoS_08 | Leishmania donovani | 1 to 566295** | 317063 | 317206 | 144 |
| **LdoS_08 | Leishmania donovani | 1 to 566295** | 442058 | 442201 | 144 |
| **LdoS_08 | Leishmania donovani | 1 to 566295** | 296053 | 296183 | 131 |
| **LdoS_08 | Leishmania donovani | 1 to 566295** | 341665 | 341793 | 129 |

**Table 4: Deletions in *LdCen*^-/-^ genome chromosome #28 compared to *L. donovani* genome**

| **Reference genome** | **Start** | **End** | **Size of deletion (bp)** |
| --- | --- | --- | --- |
| **LdoS_28 | Leishmania donovani | 1 to 1194358** | 1083270 | 1083522 | 253 |
| **LdoS_28 | Leishmania donovani | 1 to 1194358** | 1084165 | 1084381 | 217 |
| **LdoS_28 | Leishmania donovani | 1 to 1194358** | 1084384 | 1084535 | 152 |
| **LdoS_28 | Leishmania donovani | 1 to 1194358** | 1079914 | 1080064 | 151 |
| **LdoS_28 | Leishmania donovani | 1 to 1194358** | 1080480 | 1080630 | 151 |
| **LdoS_28 | Leishmania donovani | 1 to 1194358** | 1084941 | 1085091 | 151 |
| **LdoS_28 | Leishmania donovani | 1 to 1194358** | 1088462 | 1088608 | 147 |
| **LdoS_28 | Leishmania donovani | 1 to 1194358** | 1084018 | 1084155 | 138 |

**Table 5: Deletions in *LdCen*^-/-^ genome chromosome #32 compared to *L. donovani* genome**

| **Reference genome** | **Start** | **End** | **Size of deletion (bp)** |
| --- | --- | --- | --- |
| **LdoS_32 | Leishmania donovani | 1 to 1593391** | 1246901 | 1247236 | 336 |
| **LdoS_32 | Leishmania donovani | 1 to 1593391** | 1465499 | 1465776 | 278 |
| **LdoS_32 | Leishmania donovani | 1 to 1593391** | 1462293 | 1462564 | 272 |
| **LdoS_32 | Leishmania donovani | 1 to 1593391** | 1452155 | 1452382 | 228 |
| **LdoS_32 | Leishmania donovani | 1 to 1593391** | 902802 | 902996 | 195 |
| **LdoS_32 | Leishmania donovani | 1 to 1593391** | 1469500 | 1469652 | 153 |
| **LdoS_32 | Leishmania donovani | 1 to 1593391** | 1445990 | 1446139 | 150 |
| **LdoS_32 | Leishmania donovani | 1 to 1593391** | 1459367 | 1459516 | 150 |
| **LdoS_32 | Leishmania donovani | 1 to 1593391** | 1453588 | 1453731 | 144 |
| **LdoS_32 | Leishmania donovani | 1 to 1593391** | 1454884 | 1455017 | 134 |
| **LdoS_32 | Leishmania donovani | 1 to 1593391** | 897298 | 897421 | 124 |

**Supplementary information**

**Table 6: Deletions in *LdCen*^-/-^ genome chromosome #10 compared to *L. infantum* genome**

| **Reference genome** | **Start** | **End** | **Size of deletion (bp)** |
| --- | --- | --- | --- |
| **LinJ.10 | Leishmania infantum JPCM5 | 1 to 547235** | 173451 | 175604 | 2154 |
| **LinJ.10 | Leishmania infantum JPCM5 | 1 to 547235** | 164159 | 165330 | 1172 |
| **LinJ.10 | Leishmania infantum JPCM5 | 1 to 547235** | 169284 | 170292 | 1009 |
| **LinJ.10 | Leishmania infantum JPCM5 | 1 to 547235** | 176156 | 177122 | 967 |
| **LinJ.10 | Leishmania infantum JPCM5 | 1 to 547235** | 209697 | 210436 | 740 |
| **LinJ.10 | Leishmania infantum JPCM5 | 1 to 547235** | 168020 | 168708 | 689 |
| **LinJ.10 | Leishmania infantum JPCM5 | 1 to 547235** | 165333 | 166011 | 679 |
| **LinJ.10 | Leishmania infantum JPCM5 | 1 to 547235** | 172808 | 173449 | 642 |
| **LinJ.10 | Leishmania infantum JPCM5 | 1 to 547235** | 168710 | 169274 | 565 |
| **LinJ.10 | Leishmania infantum JPCM5 | 1 to 547235** | 170664 | 171186 | 523 |
| **LinJ.10 | Leishmania infantum JPCM5 | 1 to 547235** | 167674 | 168013 | 340 |
| **LinJ.10 | Leishmania infantum JPCM5 | 1 to 547235** | 163114 | 163457 | 344 |
| **LinJ.10 | Leishmania infantum JPCM5 | 1 to 547235** | 172204 | 172537 | 334 |
| **LinJ.10 | Leishmania infantum JPCM5 | 1 to 547235** | 163836 | 164157 | 322 |
| **LinJ.10 | Leishmania infantum JPCM5 | 1 to 547235** | 206995 | 207244 | 250 |
| **LinJ.10 | Leishmania infantum JPCM5 | 1 to 547235** | 166019 | 166253 | 235 |
| **LinJ.10 | Leishmania infantum JPCM5 | 1 to 547235** | 170321 | 170529 | 209 |

**Table 7: Deletions in *LdCen*^-/-^ genome chromosome #16 compared to *L. infantum*** genome

| **Reference genome** | **Start** | **End** | **Size of deletion (bp)** |
| --- | --- | --- | --- |
| **LinJ.16 | Leishmania infantum JPCM5 | 1 to 698903** | 388577 | 390660 | 2084 |
| **LinJ.16 | Leishmania infantum JPCM5 | 1 to 698903** | 380839 | 382692 | 1854 |
| **LinJ.16 | Leishmania infantum JPCM5 | 1 to 698903** | 384038 | 385592 | 1555 |
| **LinJ.16 | Leishmania infantum JPCM5 | 1 to 698903** | 392355 | 393752 | 1398 |
| **LinJ.16 | Leishmania infantum JPCM5 | 1 to 698903** | 385601 | 386538 | 938 |
| **LinJ.16 | Leishmania infantum JPCM5 | 1 to 698903** | 394276 | 395161 | 886 |
| **LinJ.16 | Leishmania infantum JPCM5 | 1 to 698903** | 379625 | 380431 | 807 |
| **LinJ.16 | Leishmania infantum JPCM5 | 1 to 698903** | 391095 | 391528 | 434 |
| **LinJ.16 | Leishmania infantum JPCM5 | 1 to 698903** | 393757 | 394268 | 512 |
| **LinJ.16 | Leishmania infantum JPCM5 | 1 to 698903** | 397791 | 398051 | 261 |
| **LinJ.16 | Leishmania infantum JPCM5 | 1 to 698903** | 604410 | 604661 | 252 |
| **LinJ.16 | Leishmania infantum JPCM5 | 1 to 698903** | 397398 | 397613 | 216 |
| **LinJ.16 | Leishmania infantum JPCM5 | 1 to 698903** | 600609 | 600792 | 184 |

**Table 8: Deletions in *LdCen*^-/-^ genome chromosome #8 compared to *L. infantum* genome**

| **Reference genome** | **Start** | **End** | **Size of deletion (bp)** |
| --- | --- | --- | --- |
| **LinJ.08 | Leishmania infantum JPCM5 | 1 to 495393** | 486721 | 487629 | 909 |
| **LinJ.08 | Leishmania infantum JPCM5 | 1 to 495393** | 307552 | 307841 | 290 |
| **LinJ.08 | Leishmania infantum JPCM5 | 1 to 495393** | 298981 | 299248 | 268 |
| **LinJ.08 | Leishmania infantum JPCM5 | 1 to 495393** | 304433 | 304691 | 259 |
| **LinJ.08 | Leishmania infantum JPCM5 | 1 to 495393** | 260017 | 260200 | 184 |

**Table 9: Deletions in *LdCen*^-/-^ genome chromosome #28 compared to *L. infantum*** genome

| **Reference genome** | **Start** | **End** | **Size of deletion (bp)** |
| --- | --- | --- | --- |
| **LinJ.28 | Leishmania infantum JPCM5 | 1 to 1163438** | 512611 | 513528 | 918 |
| **LinJ.28 | Leishmania infantum JPCM5 | 1 to 1163438** | 1162093 | 1162351 | 259 |
| **LinJ.28 | Leishmania infantum JPCM5 | 1 to 1163438** | 513531 | 513729 | 199 |
| **LinJ.28 | Leishmania infantum JPCM5 | 1 to 1163438** | 464936 | 465128 | 193 |

**Table 10: Deletions in *LdCen*^-/-^ genome chromosome #32 compared to *L. infantum*** genome

| **Reference genome** | **Start** | **End** | **Size of deletion (bp)** |
| --- | --- | --- | --- |
| **LinJ.32 | Leishmania infantum JPCM5 | 1 to 1547509** | 409634 | 410323 | 690 |
| **LinJ.32 | Leishmania infantum JPCM5 | 1 to 1547509** | 351507 | 351741 | 235 |
| **LinJ.32 | Leishmania infantum JPCM5 | 1 to 1547509** | 896166 | 896370 | 205 |
